# A consensus surface activation marker signature is partially dependent on human immunodeficiency virus type 1 Nef expression within productively infected macrophages

**DOI:** 10.1186/1742-4690-10-155

**Published:** 2013-12-16

**Authors:** Roshni Babu, Amanda Brown

**Affiliations:** 1 Department of Neurology, Johns Hopkins University SOM, 600 North Wolfe Street, Meyer 6-181, Baltimore, MD 21287, USA; 2 Assistant Professor, Department of Neurology, Johns Hopkins University SOM, 600 North Wolfe Street, Meyer 6-181, Baltimore, MD 21287, USA

**Keywords:** Single-cell analysis, CD14, CD69, CD86, CD68, Flow cytometry, Macrophage plasticity, Macrophage receptors, Reservoir

## Abstract

**Background:**

The high prevalence of HIV-associated comorbidities including neurocognitive disorder, high levels of residual inflammatory mediators in the plasma and cerebrospinal fluid and the resurgence of HIV replication upon interruption of antiviral treatment in HIV-1 infected individuals, strongly suggests that despite therapy HIV persists in its cellular targets which include T-lymphocytes and cells of the myeloid lineage. These reservoirs present a major barrier against eradication efforts. Knowledge of the molecular mechanisms used by HIV to modulate innate macrophage immune responses and impair viral clearance is quite limited. To explore the role of HIV in potentially modulating macrophage function through changes in protein expression, we used single-cell analyses with flow cytometry to determine whether, in unpolarized cultures, macrophage surface marker phenotype was altered by HIV infection in a manner that was independent of host genetic background.

**Results:**

These analyses revealed that at several time points post-infection, GFP + HIV-infected macrophages were significantly enriched in the CD14+ fraction (3 to 5-fold, *p* = .0001) compared to bystander, or uninfected cells in the same culture. However, the enrichment and higher levels of CD14 on HIV expressing macrophages did not depend on the production of HIV Nef. Sixty to eighty percent of macrophages productively infected with HIV after day 28 post-infection were also enriched in the population of cells expressing the activation markers CD69 (2 to 4-fold, *p* < .0001) and CD86 (2 to 4-fold, *p* < .0001 ) but suppressed amounts of CD68 (3 to 10-fold, *p* < .0001) compared to bystander cells. Interestingly, there was no enrichment of CD69 on the surface of HIV producing cells that lacked Nef or expressed a variant of Nef mutated in its SH3-binding domain.

**Conclusions:**

These findings suggest that HIV actively regulates the expression of a subset of surface molecules involved in innate and inflammatory immune signaling in primary human macrophages through Nef-dependent and Nef-independent mechanisms acting within productively infected cells.

## Background

While in vivo evidence using macaque animal models has supported the concept of the macrophage as a HIV reservoir, similar studies in humans are hampered by the need to use invasive means to extract tissue bound macrophages from tissues and hence, far less is known about the phenotype and turnover of these cells when they are actively replicating HIV or harbor latently infected viral genomes. Viral dynamics studies have suggested that the second phase of HIV decay during combination antiretroviral therapy (cART) is due to the turnover of macrophages [1,2], but other studies have disputed this notion [3]. The ability to purify and quantify by cell sorting, and study the nature of latently infected human macrophages would benefit from a reliable donor-independent cell surface marker signature that could distinguish these cells from productively infected macrophages.

Bulk analyses of infected macrophages have been limited in their ability to discriminate whether changes in surface marker expression occurred exclusively on the productively infected cells, on the bystander uninfected macrophages, or on both populations. Recombinant HIV fluorescent reporter viruses have been in widespread use although constructs that express all of the viral genes and replicate in macrophages were developed later. There is an urgent need to better understand in the cART era, the molecular mechanisms of HIV-mediated immune activation as well as the role of macrophages as reservoirs. As the productively infected cell is marked by a fluorescent marker, HIV reporter viruses can be useful in this regard in determining whether HIV acts directly and/or through the induction of soluble factors to dysregulate macrophage function. In this study, we used monocyte-derived human macrophages (MDM) generated from normal donors differing in genetic background and susceptibility to HIV, infected with recombinant macrophage-tropic GFP reporter viruses to determine, in a longitudinal fashion, the surface marker phenotype of the subpopulation of MDMs in which the virus replicates. Additionally, the phenotype of the bystander MDM, which are the uninfected cells in the HIV exposed culture that do not express HIV, and mock-infected cultures was also examined in parallel. We found that HIV-1 preferentially replicates in MDM with an activated phenotype characterized by the expression of CD14, CD69, CD86 and low levels of CD68. Moreover, infection with HIV-GFP reporter viruses that either lack Nef or expressed a mutated form of Nef in its SH3-binding domain suggested that Nef modulates the expression of CD69 on the surface of infected macrophages. In contrast, the enrichment of productively infected MDM expressing CD14 and CD86 and suppressed levels of CD68 did not depend on HIV Nef.

## Results

### Characterization of monocyte populations isolated by gradient density centrifugation

**Figure 1:**
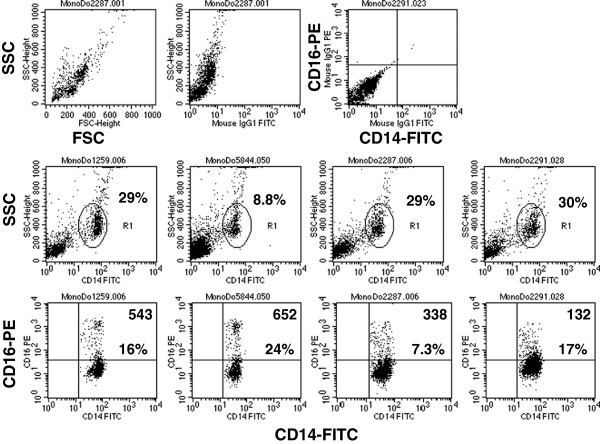
**Analysis of monocyte CD14**/**CD16 subpopulations in density gradient purified monocytes.** Ficoll purified buffy coats from normal blood donors were subjected to a second percoll gradient to enrich for monocytes and analyzed by flow cytometry for CD14/CD16 subpopulations. (Top row): The forward (FSC) and side scatter (SSC) of leukocytes after gradient purification and gating with the isotype controls are shown. (Second row): The CD14 fraction of four different donors plotted against SSC is shown and the percentage of CD14+ monocytes is given. (Bottom row): The CD14/CD16 population of four different donors is shown. The percentage of CD14 + CD16+ monocytes is given and the mean fluorescent intensity is indicated in the top right corner of each panel.

**Figure 2:**
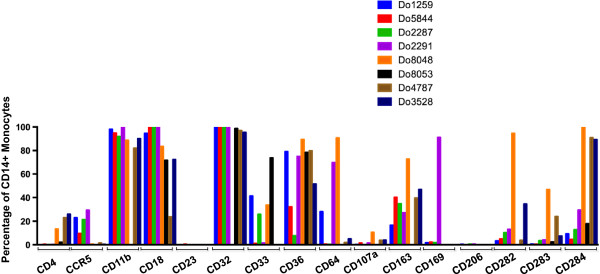
**Variable expression of typical receptors on CD14**+ **monocytes from normal blood donors.** Monocytes were double-labeled for CD14 and the indicated markers and the percentage of positively labeled cells in the CD14+ fraction was quantified by flow cytometry. Two of the donors, Do4787 (brown) and Do3528 (deep blue) were used in the longitudinal analyses of HIV-infected macrophages (Figure 3).

To ascertain whether the monocytes used in our studies reflected the previously reported heterogeneity seen in these cells, we performed flow cytometry for a panel of known monocyte/macrophage cell surface molecules on monocytes purified by density gradient centrifugation from eight normal blood donors. First, to identify the monocyte population, cells were costained with CD14. The CD14+ population was identified as gate 1 against side-scatter and then analyzed for the second marker (Figure 1). As has been reported, the percentage of monocytes in peripheral blood as well as the well-known CD14/CD16 subpopulations can vary significantly between healthy donors. In the donors analyzed, the monocyte fraction varied from 8.8-30% (Figure 1). The purity of the CD14+ fraction after gating out any CD3+ cells was 92-99%. The percentage and intensity of monocytes expressing the Fc receptor CD16 varied with the donor ranging from 7.3-24% of the CD14+ cells (Figure 1). Differences in the level of CD16 as detected in the mean fluorescent intensity (MFI) ranged from a low of 132 to a high of 652 and reflected the variation in the number of CD14 + CD16^lo^ and CD14+ CD16^hi^ monocytes among the donors, (Figure 1). While the HIV receptor CD4 and CCR5 coreceptor were detected at low (0-2%) to moderate levels (13-26%), in contrast, integrins CD11b and CD18 and the Fc receptor CD32 were abundantly expressed (72-99%) on most donors (Figure 2). The levels of Fc receptor CD64 varied markedly (0.28-91%) depending on the donor monocytes (Figure 2). The levels of CD33, a sialoadhesion expressed by monocytes and macrophages and recently shown to be associated with late-onset Alzheimer’s disease and the scavenger receptor CD36 varied between donors (1-74%, CD33; 8-89%, CD36) (Figure 2). Lysosomal-associated membrane protein-1 (LAMP-1 or CD107a), a lysosomal protein found on the surface of activated monocytes [4] was expressed at no or very low levels (0.2-4%) on most donors except for one (10%) (Figure 2). Interestingly, the marker for alternatively activated monocytes/macrophages, CD163 was detected at appreciable levels (16-73%) on most donors, while in contrast CD206 levels were absent or minimal (0-.66%) (Figure 2). Out of the four donors analyzed for this marker, CD169, a sialoadhesin implicated in chronic HIV infection and disease progression [5]–[7] was highly expressed on one donor (Do2291, 5%), but at much lower levels on all other donors (1-2%) (Figure 2). Toll-like receptor TLR2 (CD282) levels were variable ranging from a low of 3-13% and high at 34-94% (Figure 2). Levels of TLR3 (CD283) on the monocyte surface was low on most donors (0.49-7%) and elevated on two other samples (24% and 47%). A similar variation in TLR4 (CD284) was detected (4-100%) again depending on the donor monocytes (Figure 2). These data suggest that the density-purified monocytes used in this study were characterized by the abundance of known monocyte/macrophage markers, which have been described for this population and that the extent of expression of specific receptors varied between normal donors.

### Infection of monocyte-derived macrophages (MDM) with the HIV-GFP reporter virus

**Figure 3:**
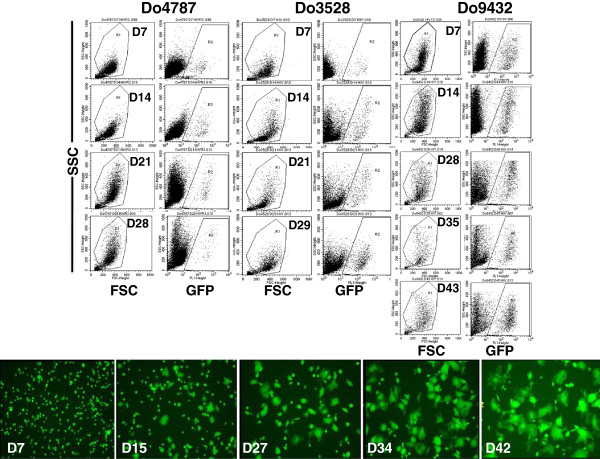
**Longitudinal analyses of monocyte**-**derived macrophages** (**MDM**) **with HIV**-**GFP.** Monocytes from three different donors, Do4787, Do3528 and Do9432 were differentiated in RPMI 1640 complete medium without the addition of any exogenous cytokines. At day 7 post-differentiation, macrophages were infected with HIV-GFP and GFP fluorescence was quantified by flow cytometry at 7–8 day intervals. The forward (FSC) and side scatter (SSC) and GFP + versus SSC, is shown in the panels. Fluorescent images of representative HIV-GFP infected MDM cultures at the indicated time points are shown.

**Figure 4:**
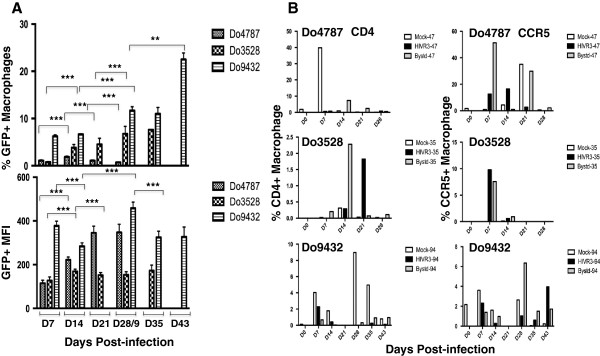
**Quantification of GFP** + **HIV**-**infected macrophages and HIV receptor and coreceptor expression. (A)** The percentage and mean fluorescent intensity (MFI) of GFP + macrophages for each donor (Do4787, Do3528 and Do9432) at the indicated time points after infection as quantified by flow cytometry is shown (mean and standard deviation). **(B)** The percentage of the macrophages expressing the HIV receptor CD4 and coreceptor CCR5 was determined at the indicated time points. ***p < .0001.

The current state-of-the-art to study human macrophage-HIV interaction is through the in vitro differentiation of donor blood monocytes and infection with macrophage-tropic viral isolates. Relatively few studies have characterized the human macrophage subsets that arise during in vitro culture or investigated whether HIV-1 replicates selectively in different populations. Two studies have reported that HIV replication in macrophages polarized to the M1- or M2-type was significantly reduced compared to unpolarized conditions [8,9]. To determine the phenotype and frequency of M1- and M2-type macrophages in our culture model and whether HIV had a preference to replicate in a specific macrophage subpopulation, we infected three different donor MDMs with the recombinant macrophage-tropic HIV_SF162_R3Nef^+^GFP reporter virus, referred to as HIV-GFP [10,11]. The study design allowed us to determine whether host genetic background had any influence on macrophage surface phenotype as well as to assess any impact on the subpopulation in which HIV replicates. Moreover, we could analyze and quantify the macrophages in which HIV actively replicated, via GFP + single-cell analyses, as well as characterize the bystander MDM, which do not express HIV, but are present in the infected culture, something not possible with previous studies using bulk culture methods. We will refer to the mock-treated, HIV-GFP+, and bystander cells as “MDM groups”. The expression of GFP indicates active transcription of the viral genome and was followed by microscopy and quantified longitudinally by flow cytometry. In infected cultures, both MDM granularity and cell size increased with time in culture, a phenomenon that is accelerated by HIV-mediated enhancement of multinucleated giant cell formation (Figure 3). The percentage and level of GFP expression in the live cell population was quantified at 7–8 day intervals. Donor 4787 (Do4787) had a peak level of GFP + MDM of nearly 2% (1.87 +/- 0.16%, n = 10) at day 14 post-infection (pi), while Do3528 peaked at day 28 pi with about 7% GFP + (7.18 +/- 1.1%, n = 10), and Do9432 increased significantly (*p* < .0001, n = 10) from D29 pi (10.84 +/- 1.2%) to peak at day 43 pi at 23% GFP + (22.71 +/- 1.5%) (Figure 4). For Do3528 and Do9432 viral spread as indicated by an increase in the number of GFP + MDM with time, continued significantly (*p* < .0001, n = 10) throughout the culture period in contrast to Do4787 where spread was more limited and occurred up until day 21 pi (Figure 4). However, GFP mean fluorescent intensity (MFI) increased significantly (*p* < .0001, n = 10) until day 21 pi for Do4787 (335 +/- 44.8) (Figure 4). For Do3528 the GFP MFI increased significantly from day 7–14 pi (128 +/- 15.4 to 168 +/- 10.3) then dropped at day 21 pi (146.5 +/- 14.5, n = 10) and did not vary much for the remainder of the culture period (Figure 4). The GFP MFI for Do9432 peaked at day 29 pi, (451 +/- 28, n = 10) and then dropped significantly to 310 +/- 38.4 MFI and remained near this level (Figure 4).

The variation in the extent of HIV replication among these three donor MDMs reflected the genetic variation in susceptibility to HIV that has been previously reported [12]. To ascertain whether differences in receptor expression were related to the extent of viral replication seen among the donors, the levels of CD4 and CCR5 were examined. Between donors, CD4 levels varied considerably with Do4787 having high levels (40%) at early time points that were rapidly lost with time, a low level of 4-10%, or very minor expression of <1% with Donors 9432 and 3528, respectively (Figure 4). In mock-treated MDM for Do9432, CD4 levels remained detectable at a low level (9% or less) throughout the culture period although the receptor was downregulated on HIV-infected and bystander MDM (Figure 4). It is known that HIV downregulates the expression of CD4 through interactions with its Env and Nef proteins [13,14]. CCR5 expression also varied with donor, but generally was typically 10% or less and waned rapidly with time in culture (Figure 4), in agreement with previous studies [15,16]. Compared to the other two donors, Do4787 expressed relatively high levels of CCR5 up to day 21 pi (38%), but was the least susceptible to HIV replication and spread (Figure 4). Hence, differences in the expression of CD4 and CCR5 between donor MDMs while likely contributing to the initial infectivity [15]–[17], did not fully account for the contrasts in the extent of replication observed. Two of the donor monocytes used in the longitudinal analyses had similar profiles of cell surface markers before differentiation except for lower CD18 and CD282 and higher CD283 levels on Do4787 compared to Do3528, the donor with greater susceptibility to HIV infection (Figures 1 and 2).

### Longitudinal analyses of macrophage surface receptor expression on mock-treated, HIV-GFP + and bystander subpopulations

**Figures 5:**
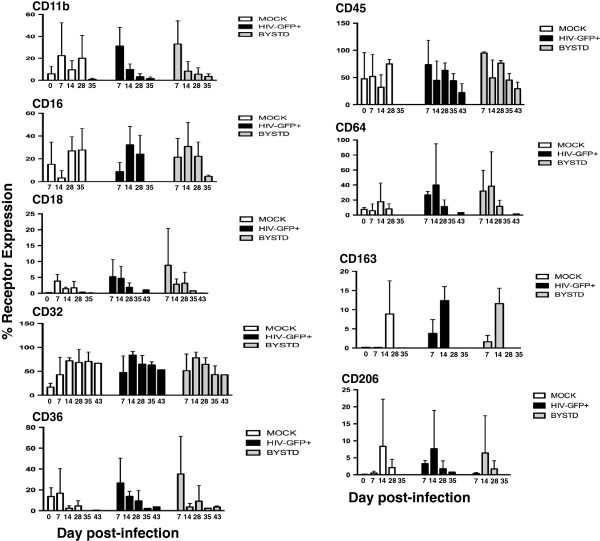
**Longitudinal analyses of surface marker expression in mock**, **HIV**-**GFP infected and bystander macrophages.** Mock- and HIV-GFP infected macrophages were harvested at 7–8 day intervals and stained for flow cytometry for the indicated markers. The day 0 time point is given only for the Mock sample and represents the day of infection. The uninfected or GFP negative cells in the HIV-GFP culture were designated as the bystander MDM (BYSTD). For each group the combined mean percentage of positively stained cells and standard deviations from all three donors is shown. Statistical comparisons were determined by one-way ANOVA with Tukey’s test for multiple comparisons and significance of *p* < .05. For the receptors shown, no significant differences were found. For the day 43 pi time point, values shown are from a single donor, Do9432.

To determine whether HIV replication alters, in a donor-independent way, the expression of macrophage subpopulations as defined by the presence of known macrophage surface markers, longitudinal analyses by flow cytometry were performed. The culture conditions used in this study did not involve differentiation under polarizing conditions and sera were tested empirically for their ability to support macrophage differentiation. No exogenous factors were added. Day 0 represented seven days after the differentiation of monocytes into MDM and the time point at which the cells were infected with HIV-GFP. Side and forward scatter was used to gate out any dead cells. Macrophages in the live cell fraction (gate R1) were analyzed by side scatter and in the FL1 channel (GFP) to identify the infected (GFP+, gate R2) and bystander (GFP-, gate R3) cells. The gated populations were then analyzed in the FL2 (PE), FL3 (PerCP) or FL4 (APC) channels for the appropriate antibody as shown in Figure 5.

Integrin receptor CD11b was expressed on 10% or less on all MDM donors at day 0 (Figure 5). By day 7–14 pi, the percentage of CD11b + MDM increased in all donors over a wide range (5-55%), but the differences were not significant. The peak in CD11b expression was then followed by a precipitous decline, but remained detectable at a very low level on two of the three donors during the entire culture period (Figure 5). Beta-2 integrin, CD18, which can complex with CD11b forming the CR3 receptor, displayed a low level of expression of 5-20% on all subpopulations at day 7 pi with no significant differences between groups (Figure 5). After day 7 pi, CD18 expression declined on all MDM groups (Figure 5).

The protein tyrosine phosphatase, CD45 was abundantly expressed (50-100%) on all donor MDMs at day 0 and remained high on all groups in all donors until day 28 pi after which the receptor became undetectable on mock-treated MDM, but remained elevated at 20-40% on HIV-infected and bystander MDM in Do9432 (Figure 5). There were no statistically significant differences between groups.

Next, the expression of the IgG receptors, which are important for immunological responses, was examined. At day 0, the expression of the low affinity IgG receptor CD16 (FcγR1) varied from 18-38% depending on the donor (Figure 5). CD16+ levels increased to 30-50% after day 7 pi on all MDM groups until day 14 pi before gradually decreasing (Figure 5). There were no significant differences in CD16+ levels between MDM groups (Figure 5).

The FcγRII, CD32, which mediates phagocytosis and oxidative burst, increased in expression from day 0 to day 7 from 20% up to as much as 80% on some donors and remained at high levels on all donors and subpopulations throughout the culture period (Figure 5). There were no significant differences in CD32 levels between MDM groups during the culture period. CD64 expression was low at less than 10% at day 0 on all donor MDMs and was depending on the donor, upregulated thereafter particularly on HIV + and bystander MDM however, the differences between groups were not significant. The rapid decrease in CD64 levels seen with time after day 14 pi for some donors did not appear to be associated with HIV infection as the same kinetics were observed on mock-treated MDM (Figure 5).

CD36, the collagen type I or thrombospondin receptor was, depending on the donor, expressed at moderate levels of 20-70% between day 0–7 pi and then was rapidly downregulated on all MDM groups, but no significant differences between groups were detected (Figure 5). The mannose receptor, a marker of M2-type- or alternatively activated macrophages, CD206 was upregulated on all MDM groups after day 7 pi reaching levels as high as 23% in some cases, but there were no significant differences between groups (Figure 5). At day 14 pi, in the HIV-infected and bystander groups and day 28 in the mock-infected group, CD206 levels fell rapidly thereafter.

Another marker of alternatively activated macrophages, CD163 was largely absent at day 0 on all MDM groups despite being present on their respective monocytes at levels of 45-50% (Figures 5 and 2). An increase in CD163 expression of 2-8% was first detected at day 7 pi in the HIV-infected and bystander MDM groups (Figure 5). At day 14 pi levels increased to 8-18% on all MDM groups and the differences were not significant. Thereafter, CD163 expression declined to undetectable on all subpopulations and was not quantified at later time points (Figure 5). Other M2-type receptors such as CD209, CD360 (IL-21R), and M1-type surface molecules such as CD127, CD215 and HLA-DR were either not detected or seen at negligible levels in this MDM culture model (data not shown).

### Surface markers selectively modulated on HIV GFP + infected macrophages

**Figure 6:**
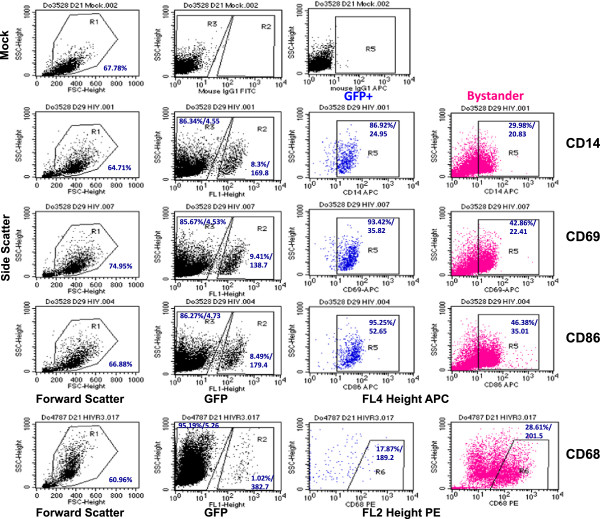
**Flow cytometric analyses for CD14**, **CD69**, **CD86 and CD68 expression on the surface of HIV**-**GFP infected MDM.** Representative scatter plots are show for the MDM donor data summarized in Figure 7. *Left column*. Forward (FSC) and side scatter (SSC) for mock-infected and HV-GFP gated for the live cells (R1) is shown and the percentage of live cells indicated. *Second column from left*. MDM in the live cell gate (R1) were analyzed by SSC and in the FL1 channel to identify the GFP + HIV-infected MDM (gate R2) and the GFP- or bystander cells (gate R3). Mock-infected MDM stained with isotype control antibodies were used for gating as indicated in the top row. The cells in gates R2 and R3 were then analyzed in the FL4 channel for the indicated surface markers. The percentage of positive cells and the mean fluorescent intensity of staining are given.

**Figure 7:**
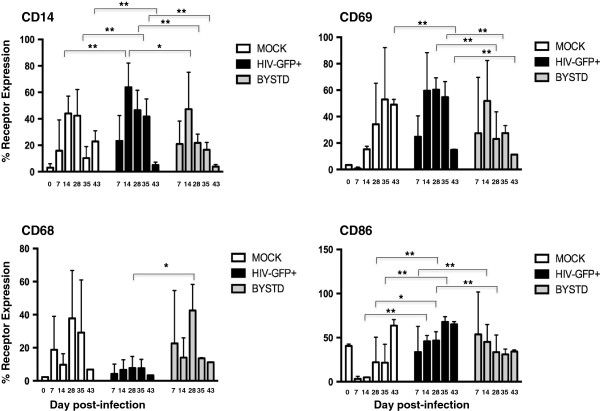
**Surface markers expression on mock**, **HIV**-**GFP** + **or bystander macrophages from Do4787**, **Do3528 and Do9432.** Mock- and HIV-GFP infected macrophages were harvested at 7–8 day intervals and stained for flow cytometry for CD14, CD68, CD69, and CD86. The day 0 time point is given only for the Mock sample and represents the day of infection. The uninfected or GFP negative cells in the HIV-GFP culture were designated as the bystander MDM (BYSTD). For each group the combined mean percentage of positively stained cells and standard deviations from all three donors is shown. Statistical comparisons were determined by one-way ANOVA with Tukey’s test for multiple comparisons and significance of *p* < .05.

In contrast to the macrophage markers analyzed above, a different pattern was observed for CD14, CD69, CD86 and CD68. Representative scatter plots are shown in Figure 6. The percentage of CD14+ macrophages at day 0 to day 14 in culture varied with donor but generally started out low at 0.1-6% and then dramatically increased up to 80% on some MDM groups (Figure 7). At day 14 pi the difference in CD14 levels between HIV-infected (63.8 +/- 18.3, n = 17) and bystander MDM (47.3 +/- 27.9, n = 18) (*p* = .047) approached significance (Figure 7). After day 21 pi, the level of CD14+ on HIV-infected MDM (46.5 +/- 15.1, n = 16; day 28; 41.7+/-13.3, n = 12) significantly exceeded the levels on bystander cells (21.8 +/- 6.71, n = 16 day 28; 16.5 +/-5.62, n = 11; day 35; *p* = 1.4e-6, day 28; *p* = 9.2e-6, day 35) (Figure 7). CD14+ macrophages were significantly greater in the HIV-infected group (63.8 +/- 18.3, n = 17, day 14; 41.7 +/-13.3, n = 12, day 35) compared to mock-infected cells (44 +/- 13.1, n = 18, day 14; 10.3 +/- 8.67, n = 10, day 35) at Day 14 and 35 pi (*p* = .0007, D14; *p* = 3e-6, D35, n = 7), but lower in Do9432 at D43 pi (mock: 22.8 +/- 8, HIV: 5 +/- 2.13, *p* = .0009, n = 6) (Figure 7).

In all three donors there was no or low-level expression (3.5%) of CD69 at day 0 (Figure 7). However, by day 28 and 35 pi, CD69 expression was significantly higher on HIV-GFP + MDM (60.4+/-8.87, n = 6, day 28; 54.8 +/-11.6, n = 4, day 35) compared to bystander cells (23.11 +/- 20.5, n = 6, day 28; 27.6 +/- 5.6, n = 4; day 35; *p* = .002, day 28; *p* = 0.005, day 35) (Figures 6 and 7). Abundant expression of CD69 was maintained on HIV-GFP + MDM for all donors at all subsequent time points (mean std, *p* = .0002, n = 3, day 28 pi; mean std, *p* = .005, n = 3, day 43 pi). With Do9432, in contrast to the other two donors, CD69 expression was also high on mock-treated MDM suggesting that this donor possessed an intrinsic high level of activation (Figure 7).

The costimulatory marker CD86 was by day 14 pi significantly induced on HIV-GFP + MDM (45.8 +/- 6.56, n = 5) compared to mock-treated cells (4.92 +/- 0.39, n = 3, *p* = 4.5e-5) and was sustained at day 28 (mock: 22 +/- 28.5, HIV: 46.7+/- 9.95, *p* = .026, n = 9) and day 35 pi (mock: 21.5 +/- 20.9, HIV: 67.7+/- 5.89, *p* = .005, n = 4) (Figure 7). CD86 levels were significantly higher on HIV-GFP + MDM compared to bystander MDM beginning at day 35 pi (HIV: 67.7+/- 5.89, Bystander: 33.5 +/- 19.4, n = 4) and a similar trend was observed in Do9432 at day 43 pi HIV: 65.2 +/- 2.92, Bystander: 34.1 +/- 1.77, n = 2) (Figures 6 and 7).

Expression of CD68, a member of the scavenger receptor family was variable depending on the donor and ranged from a low of 3% to a high of 66% on mock-treated and bystander MDM on all donors from day 7 up to day 35 pi, however the differences were not significant (Figure 7). Compared to levels on bystander MDM at day 28 pi, CD68 was significantly downregulated on HIV-GFP + MDM (HIV: 7.74 +/- 6.98, *p* = .01, n = 4; mock: 37.7+/- 28.9, n = 4), and remained low on HIV-infected MDM at all time points (Figure 7).

Based on analyses of the mean fluorescent intensity (MFI) for CD14, significantly higher density of this receptor was detected on HIV-GFP + MDM compared to bystander cells at day 14 after infection (Table 1). Similarly, the density of CD86 on HIV-GFP + MDM exceeded that on bystander cells at day 21–28 post-infection (Table 1). No other significant differences in MFI between the HIV-GFP + and bystander groups were seen at other time points or with CD69 or CD68. These findings suggest that, irrespective of their overall density on the surface, CD14, CD69, CD86 and CD68^low^ represent a potential consensus surface marker phenotype of productively HIV-infected macrophages.

**Table 1:** **Comparison of marker mean fluorescent intensity on Do4787**, **Do3528**, **and Do9432 macrophages**

**Marker**	**CD14**			**CD69**			**CD86**			**CD68**		
**Sample**	**Mock**	**HIV**-**GFP+**	**Bystander**	**Mock**	**HIV**-**GFP+**	**Bystander**	**Mock**	**HIV**-**GFP+**	**Bystander**	**Mock**	**HIV**-**GFP+**	**Bystander**
Day 0 Avg	39.12 (n = 8)	n/a	n/a	48.21	n/a	n/a	68.57	n/a	n/a	68.66	n/a	n/a
Day 0 SD	32.61	n/a	n/a	n/a	n/a	n/a	n/a	n/a	n/a	n/a	n/a	n/a
*Significance p* < .*05*										n/a		
Day 7 Avg	59.81 (n = 9)	39.15	32.28	54.49	44.38	29.24	53.85 (n = 4)	30.59	43.45	47.69	20.95	16.22
Day 7 SD	42.48	19.45	9.19	n/a	n/a	11.01	68.1	24.03	18.78	3.555	n/a	n/a
*Significance p* < .*05*				n/a	n/a	n/a						
Day 14 Avg	52.98 (n = 18)	**49.2**	**37.42**	56.03 (n = 3)	49.41	33.29	86.91 (n = 3)	49.99	34.05	71.86	67.91	53.59
Day 14 SD	24.77	**20.93**	**8.28**	21.36	22.33	15.44	61.77	17.57	8.67	2.44	22.27	8.62
*Significance p* < .*05*		*										
Day 21–28 Avg	74.56 (n = 17)	66.23	52.9	63.53 (n = 6)	59.91	72.67	114.32 (n = 9)	**74.33**	**33.49**	97.86	128.51	96.52
Day 21–28 SD	28.54	24.59	15.98	32.27	32.81	44.41	71.02	**26.29**	**25.85**	54.05	43.18	79.6
*Significance p* < .*05*								*				
Day 29–35 Avg	66.64 (n = 10)	50.81	35.91	50.76 (n = 3)	46.81	41.67	112.29 (n = 4)	86.41	60.91	62.45	100.37	66.34
Day 29–35 SD	23.99	24.43	12.39	19.88	16.75	12.28	57.61	34.87	7.52	36.22	82.29	12.36
*Significance p* < .*05*												

Data were analyzed in unpaired *t* tests with Holm-Sidak correction for multiple comparisons and significance at p < .05 using GraphPad Prism 6 software. Significant differences between the HIV-GFP+ and bystander groups are indicated in bold and by an asterisk.

### Active modulation of macrophage surface phenotype by HIV Nef-dependent and independent mechanisms

**Figure 8:**
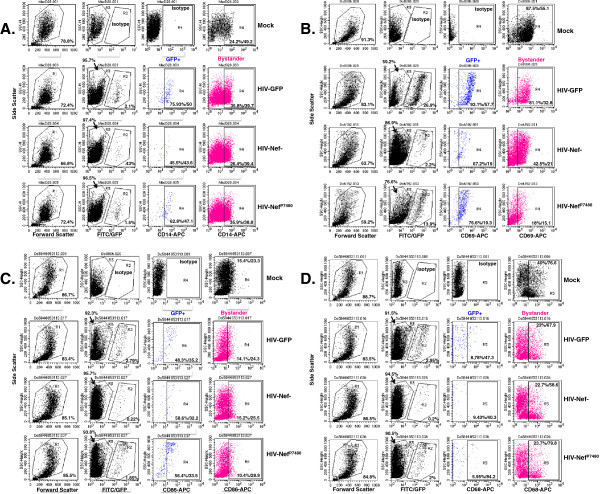
**Flow cytometric analyses for CD14**, **CD69**, **CD86 and CD68 expression on the surface of MDM infected with the HIV**-**Nef+, HIV-Nef- and HIV-Nef**^**P7480 **^**GFP reporter viruses.** Representative scatter plots are show for the MDM donor data summarized in Figure 9. *For each panel A-D: Top row*: mock-infected MDM stained with isotype controls or CD14-APC antisera. *Left column*. Forward (FSC) and side scatter (SSC) for the indicated infected MDM gated for the live cells (R1) is shown and the percentage of live cells indicated. *Second column from left*. MDM in the live cell gate (R1) were analyzed by SSC and in the FL1 channel to identify the GFP + HIV-infected MDM (gate R2) and the GFP- or bystander cells (gate R3). The percentage of GFP + cells is indicated in the lower right corner and of bystander cells in the upper left as indicated by the arrow. *Third and fourth columns from left*. The cells in gates R2 and R3 were then analyzed in the FL4 channel for CD14 (Figure 8A), CD69 (Figure 8B), CD86 Figure 8C and CD68 (Figure 8D). The percentage of positive cells and the mean fluorescent intensity of staining are given.

**Figure 9:**
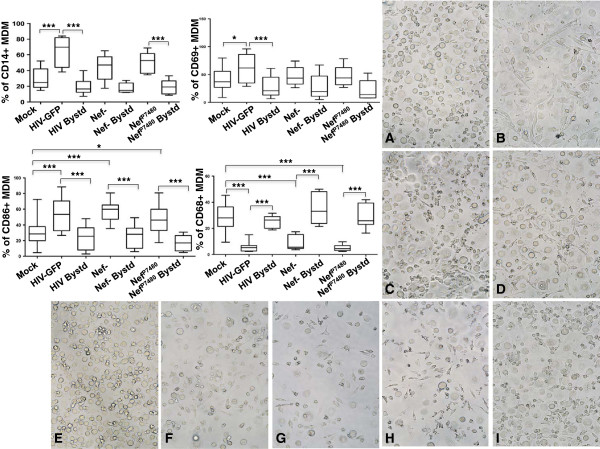
**HIV Nef**-**dependent and Nef**-**independent modulation of HIV**-**GFP macrophage cell surface phenotype.** Nine donor MDM infected with HIV-GFP, HIV-GFP-Nef^-^, HIV-GFP-Nef P7480, or mock-treated were harvested between day 15–28 post-infection and analyzed by flow cytometry with anti-CD14-PE and CD69-APC or CD68-PE and CD86-APC as shown in the panels at the upper left. Statistical comparisons were determined by one-way ANOVA with Tukey’s test for multiple comparisons and significance of *p* < .05, *** < .0001. Images of macrophage monolayers of nine donors at day 15 pi is shown. Figure 9A-I.

Our results suggested that CD14, CD68, CD69 and CD86 cell surface expression is altered in macrophages that are actively replicating HIV and not on the bystander uninfected cells. Alternatively, it was possible that MDM expressing the appropriate levels these receptors were the preferred target cells of HIV and that there was no active modulation by infection. To distinguish among these possibilities and expand on the analyses with the initial three donors, we used nine different donor MDMs infected with HIV-GFP, with a variant lacking Nef, or expressing a mutant form of Nef inactivated in the SH3-binding domain [10,18]. Nef, which is required for pathogenesis in vivo and for high-level replication in primary cells [19]–[22], has been shown to downregulate a number of cell surface molecules important for immune surveillance and antigen presentation [13,14,23,24]. MDM were harvested at day 15 or day 21–28 pi and stained with fluorescently labeled antibodies against CD14, CD69, CD86 and CD68 and analyzed by flow cytometry. Representative scatter plots for each of these markers are shown in Figure 8A-D. The round, fried-egg appearing macrophages were the predominant morphology seen in the majority of these cultures, however four of the nine donors shown had significant numbers of spindle-shaped macrophages (Figure 9 B, D, G and H). Despite this heterogeneity, a significantly higher percentage of HIV-Nef + infected MDMs (HIV-GFP) expressed CD14 compared to mock-infected (*p* = .0001) and HIV-bystander MDMs (mock: 29.4 +/-13.3; HIV-GFP Nef+: 64.6 +/- 19.1, HIV-Nef + Bystander: 19.5 +/- 10.5, *p* < .0001, n = 9, Figures 8A and 9). There was a trend of a higher number of CD14+ MDM infected with the Nef- mutant compared to the Nef-Bystander cells (Nef-: 44.2 +/- 17.4, Nef-Bystander: 17.7+/- 6.5, *p* = .057, n = 5), while a significant difference in the percentage of CD14+ macrophages productively infected with the Nef P7480 variant compared to its bystander population was detected (Nef P7480: 49.8 +/- 13.7, Nef P7480-Bystander: 18.8+/- 9.5, *p* = .015, n = 5, Figures 8A and 9). These results suggested that Nef is not required for the enrichment of CD14 on productively infected MDM.

The percentage of MDM expressing CD69 was significantly higher on HIV-GFP Nef + MDM compared to mock and HIV-Nef + bystander cells (mock: 39.6 +/- 18.9, *p* = .022, n = 17; HIV: 61.7 +/- 25.5; HIV-Nef + Bystander: 26.4 +/- 17.8, *p* < .0001, n = 17, Figures 8B and 9). In contrast, CD69 surface expression was not differentially enriched on MDM infected with the Nef- or Nef P7480 mutants compared to their respective bystander cells (Nef-: 46.7 +/- 16.3, Nef-Bystander: 29.2 +/- 21.4, n = 12; Nef P7480: 48 +/- 18.2, Nef P7480-Bystander: 21.9 +/- 18, n = 6) (Figures 8B and 9). These data suggest that a functional Nef protein with an intact SH3-binding domain expressed in the productively infected macrophage is required for the enrichment of CD69 on the cell surface.

An increased percentage of CD86+ MDM in the HIV-GFP Nef + compared to mock and HIV-Nef + bystander MDM was confirmed on the larger donor set (mock: 31.8 +/- 13.1; HIV: 51.4+/- 18.6; HIV-Bystander: 25.48 +/- 14.58; Nef-: 57.3 +/- 12.9; Nef-Bystander: 25.9 +/- 13.8; Nef P7480: 48.5 +/- 19.6; Nef P7480-Bystander: 16.5 +/- 9.7; *p* < .0001, n = 20 Figures 8C and 9). Significantly enrichment of CD86 was also found on MDM infected with the Nef- and Nef P7480 variants compared to their respective bystander cells, suggesting that Nef is not required for the upregulation of this receptor (Figures 8C and 9).

Strong suppression of CD68 levels were seen on MDM infected with HIV-GFP Nef+, Nef- and the Nef P7480 variants compared to their respective bystander MDM and mock-treated cells (mock: 28.2 +/- 11.7; HIV: 5.75 +/- 3.64; HIV-Bystander: 25 +/- 4.6, n = 13; Nef- 8.64 +/- 5.8; Nef-Bystander 35.1 +/- 11.5, n = 6; Nef P7480 5.11 +/- 2.61; Nef P7480-Bystander, 29.4 +/- 8.77; n = 14, p < .0001, Figures 8D and 9). These results suggest that Nef does not play a role in modulating the surface levels of this receptor and hence, additional HIV-mediated mechanisms in productively infected macrophages are involved in the suppression of CD68 expression.

## Discussion

Macrophages are exquisitely sensitive to their microenvironment having the ability to respond to innate immune signals and modify their gene expression profiles [18]. In this study exogenous cytokines were not used to polarize macrophages to the M1- or M2-type subpopulations that have been descried [8]. Instead, we wanted to determine whether HIV infection could promote macrophage gene expression changes along M1-, M2- or some other type of pathway. While the majority of donor macrophages upon differentiation exhibited a round morphology with a fried-egg appearance, 80% of donors also contained significant numbers of spindle-shaped cells. This is of note as it has been reported that monocytes differentiated under non-polarized conditions exhibit a round morphology and possess M2-like characteristics such as CD163 and TGF-β2 expression while spindle-shaped macrophages had a pro-inflammatory profile [25]. The majority of the macrophages in the herein described cultures had a round morphology and did not express CD163, highlighting variation that is observed in different culture models. The expression of M2-type receptors CD163 and CD206 was very low in our culture model in agreement with a study by Porcheray et al. [26]. Lacking the ability to quantify receptor levels separately on infected and bystander macrophages, the latter study reported that CD14 levels were dramatically decreased on all macrophages in HIV-infected cultures with time [26]. CD86, which was highly expressed on HIV-infected macrophages, and other receptors including CCR7, CD127, CD215 and MHC II have been described as markers for M1-type macrophages [18,27], but were absent from HIV-GFP infected cells. The HIV-infected macrophages in our culture model appear to be most related to the M1-type by the presence of CD86 and the heightened state of activation indicated by the enrichment of CD69 and CD14 on the cell surface but are clearly different from the prototypical M1-type macrophages. Moreover, our study revealed for the first time that changes in gene expression within the productively infected macrophage that impact CD14, CD69, CD86 and CD68 levels do not impact cells in the same culture, which are not expressing HIV. This suggests that alterations in the level of the latter surface molecules are not impacted by the release of soluble factors.

Indeed, the ability to analyze the cell surface of productively HIV-infected macrophages as well as the GFP negative, uninfected cells in the same culture allowed us to determine that HIV viral proteins play an active role in modulating the surface phenotype of these cells irrespective of differences in genetic background and susceptibility to HIV replication between donor macrophages. A consensus cell surface signature on macrophages that actively express HIV defined as CD14+, CD69+, CD86+ and CD68^low^ was found to be consistent on the 12 different donors used in this study. This signature is expected to be present on MDM when HIV replication is robust and to wane as viral expression decreases. CD69 was significantly increased on HIV-infected cells only when Nef was produced. Expression of Nef encoding mutations within its SH3-binding domain did not restore elevated levels of CD69 on infected macrophages, implicating this protein interaction domain in the mechanism of CD69 upregulation. An activated cellular microenvironment as found in cells at the G_1b_ to G_1/s_ boundaries allows for efficient HIV replication as the host cell factors involved in promoting reverse transcription, nuclear import and transcription are more highly expressed under these conditions [28,29]. Additionally, HIV has evolved mechanisms to infect non-dividing cells like macrophages. In this regard, Nef protein is required for high-level replication of HIV in primary macrophages and T-cells [20,22,30], for pathogenesis in several animal models of HIV infection [31,32] and in humans infected with variants lacking Nef, the disease course is very attenuated [33]. HIV Nef downregulation of CD4 [34] and selective classes of MHC class I molecules [23] from the surface of infected cells in a mechanism, which facilitates immune escape from CTL lysis [35] while providing protection from NK cell lysis, has been well characterized in the context of viral infection or primary HIV target cells and in a variety of culture models and over-expression systems. The PXXP or SH3-binding domain of Nef is required for the downregulation of MHC class I, but not CD4 [36,37] and is under strong immune selection in vivo being highly conserved in patient derived Nefs [38]. Less well characterized is Nef’s ability to upregulate CD74, and DC-SIGN, and downregulate CD206, CD1, CCR5, CD71, CD80/CD86 and CD8 [39]. In this study with single-cell analyses, we were able to analyze the surface phenotype of HIV-infected and bystander uninfected macrophages and found that in contrast to earlier reports [40,41] that CD86 while present at low levels on bystander cells, is in fact increased 2 to 4-fold on HIV-infected macrophages. As bystander cells in this study outnumbered HIV-infected macrophages from as high as 50:1 to 5:1, this phenotype could not be observed without a method to specifically quantify productively infected cells. We found that donors differed in their level of basal expression of CD86 and CD69. Do9432, which showed the highest susceptibility to HIV replication, expressed high levels of CD86 and CD69 in the absence of infection. Indeed, a correlation between CD86 levels on macrophages and HIV replication was previously reported [42].

In most HIV-infected individuals on anti-viral therapy, undetectable levels of HIV in the plasma suggest that viral replication is effectively suppressed. However, inflammatory mediators including sCD14, IL-6, IL-8, CCL2, CCL3, CXCL10, IFNγ remain readily detectable and elevated compared to uninfected individuals [43,44]. Discontinuation of anti-viral drugs results in the resurgence of HIV replication in a little as a few weeks [45] and this finding together with the persistent immune activation, confirms that cellular and tissue reservoirs remain active. Much is known about the nature of the resting CD4+ T-cell reservoir, but the same is not true of tissue macrophages. In addition, much remains to be learned about HIV-infected macrophages and their role in the innate response and engagement and activation of T-cells. CD69 is a member of the NK cell gene complex family of signal transducers and an early T-cell activation marker as its presence on T-cells is followed by CD25 and at later stages of activation by MHC class II, HLA-DR. However, comparatively little is known with regard to CD69 function on human macrophages. IFN-γ with LPS or TNF-γ can increase CD69 expression on murine macrophages and engagement of the receptor on monocytes results in the induction of calcium flux, nitric oxide and cytosolic PLA_2_ activation [46]. The ligand for CD69 remains unknown. Recent studies in CD69-KO mice suggest a non-redundant role of the receptor in the downregulation of immune responses through TGF-γ [47]. Blocking CD69 impairs oral tolerance, exacerbates arthritis as well as other autoimmune disorders by blocking the differentiation of Th17 lymphocytes [47]. Monocytes with increased expression of CD14 and CD69 have been reported in HIV-associated dementia and culture supernatants from the latter were shown to induce apoptosis in human brain aggregates [48]. More recently CD69 positive macrophages have been shown to play a role in acute lung injury [49]. In acute lung injury, as well as in mice studies of intracellular bacterial infection, the evidence points to a role for CD69 as a negative regulator of immune activation [49]–[51].

CD14 is a high-affinity GPI-linked receptor for LPS and together with TLR4 helps to activate monocytes and stimulate cytokine secretion from these cells. Recent studies have shown that CD14 is required for TLR4 transport to endosomes [52]. TLR4 levels were very low or undetectable on MDM at multiple time points. In agreement with this, recent microarray analyses by Brown et al., reported that HIV stimulates a M1-type gene transcription program in macrophages independently of TLR activation [53]. A better understanding of the regulation of CD14 signaling on HIV-infected macrophages is needed to determine how this pathogen modulates innate and adaptive host immune responses.

The central costimulatory molecule B7.2 or CD86 is mainly expressed on antigen presenting cells and plays a role, through ligation to ligand CD28 or CLTA-4, activates or suppresses immune responses respectively, on naïve or memory T-cells. CD86 was reported to be increased on T-cells in HIV infection [54,55] and Wang and Lewis showed, in agreement with our results, that HIV production correlated with CD86 expression on macrophages [42]. In this regard, signal transduction via CD86 can lead to the activation of NFγ-γ, which is a well-known enhancer of HIV replication. Other intracellular pathogens like *Toxoplasmosis gondii* show increased expression of CD86 on murine macrophages [56]. Interestingly, choroid plexus and perivascular macrophages in the brain express costimulatory molecules that are likely increased upon the entry of pathogens into the brain and under conditions of injury and/or neurodegeneration [57].

## Conclusions

Our findings suggest that monocyte-derived macrophages productively infected with HIV express CD14, CD69, CD86 and low levels of CD68 on their cell surface. The enrichment of HIV in this subpopulation of macrophages utilizes mechanisms that are independent of HIV Nef in the case of CD14, CD86 and CD68, but require Nef function to modulate CD69 surface expression. Interestingly, for the consensus surface markers identified, the release of potential soluble factors by HIV-infected macrophages does not appear to alter the surface phenotype of bystander cells. These findings illustrate how viral infection can influence host cell gene expression most likely for the purpose of usurping inflammatory signaling pathways that could potentially inhibit HIV replication. Uncovering the macrophage-ligand-receptor interactions and functions of the consensus signature at the molecular level is needed to better understand HIV modulation of innate immunity and the nature of viral persistence in macrophages.

## Methods

### Monocyte isolation, differentiation and culture

Leukopaks from healthy donors were obtained from the New York Blood Center in accordance with a study protocol NA_00030244 approved by the Johns Hopkins Institutional Review Board. The buffy coat was isolated by Ficoll-Paque density gradient centrifugation. Monocytes were subsequently enriched using a 46% percoll density gradient [58]. Monocytes were cryopreserved in RPMI1640/20% FBS/10% DMSO until further use. Approximately 1–3 x10^6^ monocytes were differentiated in T-25 flasks treated with CellBind (Corning) in RPMI1640/20% FBS/10% human AB sera/1% penicillin-streptomycin, 1% glutamax and 1 sodium pyruvate (Invitrogen) for two-three days and then switched to the same medium without human sera (RPMI1640 complete medium). No exogenous cytokines were added, but both the FBS and human sera were tested empirically for their ability to support efficient monocyte differentiation.

### Infection of human monocyte-derived macrophages with HIV reporter viruses

At seven days post-differentiation human monocyte-derived macrophages (MDM) were infected with HIV_SF162_R3 Nef + (HIV-GFP), HIV_SF162_R3 Nef- (HIV-GFP-Nef-), or HIV_SF162_R3 Nef-P7480 in RPMI1640/2%FBS overnight [10]. The next day the medium was removed and replaced with RPMI1640 complete medium. For the first two weeks the entire culture supernatant was changed every 3–4 days and thereafter, about once a week. At the indicated time point medium was removed, MDM rinsed with PBS before the addition of 1–2 ml of Accutase (Chemicon or Sigma) to detach the cells. MDM were incubated in Accutase at 37°C for 30 min-1 hr. MDM were collected and washed in PBS/2%FBS/5 mM EDTA/10 mM sodium azide for immunostaining for flow cytometry.

### Flow cytometric methods and data analyses

The fluorescently conjugated antibodies and isotype controls (IgG1 clone MOPC-21, BioLegend) were used in single or 3-color combinations: CD4 (clone OKT4, BioLegend), CD11b (clone ICRF44, BioLegend), CD14 (clone, TuK4, Invitrogen), CD16 (clone 3G8, Invitrogen), CD18 (clone 6.7, eBioscience), CD32 (clone FUN-2, BioLegend), CD33 (clone WM53, BioLegend), CD36 (clone CB38; BD Biosciences Pharmigen), CD64 (clone, 10.1, BD Biosciences Pharmigen), CD68 (clone eBioY1/82A, eBioscience), CD69 (clone CH/4, Invitrogen), CD86 (clone BU63, Invitrogen), CD127 (clone A019D5, BioLegend), CD163 (clone GHI/61, BioLegend), CD195 (clone 45549, R&D Systems), CD206 (clone 19.2, BD Biosciences Pharmigen), CD209 (clone eB-h209, eBioscience), CD282 (clone TL2.1, BioLegend), CD283 (clone TLR3.7, eBioscience), CD284 (clone HTA125, eBioscience), CD289 (clone eB72-1665, eBioscience), and CD360 (clone 2G1-K12, BioLegend). Stained cells were analyzed on a FACSCalibur using CellQuest software (BD Biosciences). For the time course studies, all three donors were harvested, stained and analyzed on the same day. The population of cells enriched for monocytes after ficoll/percoll density gradient centrifugation were identified through CD14 vs side scatter. The CD14 gate excluded any T-cell contamination as determined by anti-CD3 co-staining. Monocytes in the CD14 gate were then quantified for the second marker. For MDM, live cells were defined using forward and side scatter properties and designated in gate 1 (R1). MDM in the R1 gate were then plotted against side scatter and FL1 for GFP expression (gate 2, R2) or for the bystander cells (gate 3, R3, GFP negative). For HIV-GFP infected cultures, gates delineating the GFP + and bystander MDM were generated and analyzed separately for the expression of the indicated cell surface markers.

### Data analyses

Statistical analyses were performed using GraphPad Prism 6 to determine mean, standard deviations, and significance using multiple unpaired *t*-tests or one-way ANOVA. Significance was determined by *p* values less than 0.05 and corrected with Tukey’s test for multiple comparisons.

### Microscopy

Images of MDM monolayers were captured at the indicated time point using a Nikon E2000U inverted epifluorescence microscope or Zeiss Axiovert A1 inverted microscope. Images were processed with Adobe Photoshop and included adjusting the color balance, contrast and application of the unsharp mask filter.

## Abbreviations

cART: Combination antiretroviral therapy; MDM: Monocyte-derived macrophages; GFP: Green fluorescent protein

## Competing interests

The authors declare that they have no competing interests.

## Authors’ contributions

RB participated in the acquisition of data and reviewed the manuscript. AB participated in the acquisition, analysis and interpretation of the data and wrote the manuscript. Both authors read and approved the final manuscript.

## Authors’ information

RB current address: National Institutes of Neurological Disorders and Stroke.
